# Design and Evaluation of a Smartphone Medical Guidance App for Outpatients of Large-Scale Medical Institutions: Retrospective Observational Study

**DOI:** 10.2196/32990

**Published:** 2022-04-21

**Authors:** Kei Teramoto, Shigeki Kuwata

**Affiliations:** 1 Tottori University Hospital Yonago Japan; 2 Department of Clinical Information Management Nara City Hospital Nara Japan

**Keywords:** mHealth, outpatient clinics, electronic medical records, COVID-19, EHR

## Abstract

**Background:**

The greatest stressor for outpatients is the waiting time before an examination. If the patient is able to use their smartphone to check in with reception, the patient can wait for their examination at any location, and the burden of waiting can be reduced.

**Objective:**

This study aimed to report the system design and postintroductory outcomes of the Tori RinRin (TR2) system that was developed to reduce outpatient burden imposed by wait times before examination.

**Methods:**

The TR2 system was introduced at Tottori University Hospital, a large medical facility that accepts a daily average of 1500 outpatients. The system, which links the hospital’s electronic medical record database with patients’ mobile devices, has the following functions: (1) GPS-based examination check-in processing and (2) sending appointment notification messages via a cloud notification service. In order to evaluate the usefulness of the TR2 system, we surveyed the utilization rate of the TR2 system among outpatients, implemented a user questionnaire, and polled the average time required for patients to respond to call notifications about their turn.

**Results:**

The 3-month average of TR2 users 9 months after the TR 2 system introduction was 17.9% (14,536/81,066). In an investigation of 363 subjects, the mean examination call message response time using the TR2 system was 31 seconds (median 14 seconds). Among 166 subjects who responded to a user survey, 86.7% (144/166) said that the system helped reduce the burden of waiting time.

**Conclusions:**

The app allowed 17.9% of outpatients at a large medical facility to check in remotely and wait for examinations anywhere. Hence, it is effective in preventing the spread of infection, especially during pandemics such as that of coronavirus disease. The app reported in this study is beneficial for large medical facilities striving to reduce outpatient burden imposed by wait times.

## Introduction

For patients undergoing outpatient examination, the wait time from check-in to the start of examination restricts their activity, contributing to mental and physical stress [1-5]. SARS-CoV-2, which causes COVID-19, was first confirmed in 2019 and is spread via droplets through close contact [6-10]. Since COVID-19 is a disease that spreads via droplets, it is important to wear a mask and keep distance among people [11-15]. In large medical institutions, where many patients have a serious underlying respiratory illness, it is important to prevent in-hospital outbreaks in crowded examination rooms. In recent years, health care apps for smartphones have become widespread in the medical field [16-19]. If patients are able to check-in for their examinations and confirm wait times and queues from their mobile devices, they can avoid crowded spaces as they wait to be seen by a health care provider.

The EasyHos System, designed by Vorakulpipat et al [20], uses patient check-in data stored in hospital information system databases to send notifications to patients’ mobile devices regarding their place in the queue for examination. The EasyHos System not only made waiting more convenient for patients but also alleviated work-related burdens on hospital staff. However, it was not introduced at large-scale medical facilities since physicians usually decide the order of examination at such facilities based on the severity of the patient’s condition and the arrival of test results, precluding automated notifications of examination start times.

To address this issue, we developed the Tori RinRin (TR2) system, an examination guidance app that can be implemented at large medical facilities. In the TR2 System, a physician can send a notification to an app installed on the patient’s mobile phone to call them for examination. This notification is generated while the physician operates the electronic medical record (EMR) screen and reviews information such as the patient’s pre-examination test results and check-in queue. This system allows the patient to arrive outside the examination room just before their examination begins, thereby shortening the time spent in the waiting room. The TR2 System is also equipped with a GPS-based examination check-in function that allows patients to check-in for their examination from outside the hospital as long as they are within a 500-meter radius of the hospital. Notifications to patients’ mobile devices are sent using a cloud messaging notification function that does not depend on the type of mobile device or operating system.

The inclusion of these features in the TR2 system has enabled its introduction at Tottori University Hospital (TUH), a large medical facility that receives over 1500 outpatients a day. In this study, we aimed to retrospectively assess system design and introduction of the TR2 system to examine the performance of the system’s cloud messaging notification function and user registration during the COVID-19 pandemic in order to improve outpatient convenience.

## Methods

### Experimental Setting

This retrospective study was an analysis of existing, anonymized patient data and materials. According to clinical and epidemiological research guidelines concerning anonymous patient data, ethical review was not required.

TUH, where the TR2 system was developed and introduced, is a medical facility with 40 departments and 697 beds. TUH provides high-level advanced medical care. The TR2 system was developed and named by a research group, which includes the authors of this study who are affiliated with TUH. TUH did not provide a medical guidance application as a countermeasure for outpatient waiting time before the introduction of the TR2 system. The introduction of the TR2 system enabled patients to use an examination guidance app free of charge. The examination guidance app allows users to receive an advance notification message before the start of an examination on their smartphones indicating the expected start time. To promote use of the examination guidance app at TUH, 2 dedicated operators were stationed at the main entrance of the outpatient facility to assist patients in registering for and using the app. For patients who did not have a smartphone owing to financial or other reasons, we rented out an iPod touch with the examination guidance app installed, if desired. There was no change in the waiting order for medical examination regardless of whether the medical examination guide app was used; however, if the patient did not present to the medical examination room when they were notified via the app, the next patient was examined.

The TR2 system was introduced on a trial basis in 3 clinical departments in July 2019 and started operation in October 2019 for all 40 outpatient facilities.

From July 2020, to prevent nosocomial transmission of COVID-19, TUH has used posters and pamphlets to promote the use of its examination guidance app to help patients maintain distance from others as they wait within the hospital for their examinations.

### Outline of the TR2 System

**Figure 1:**
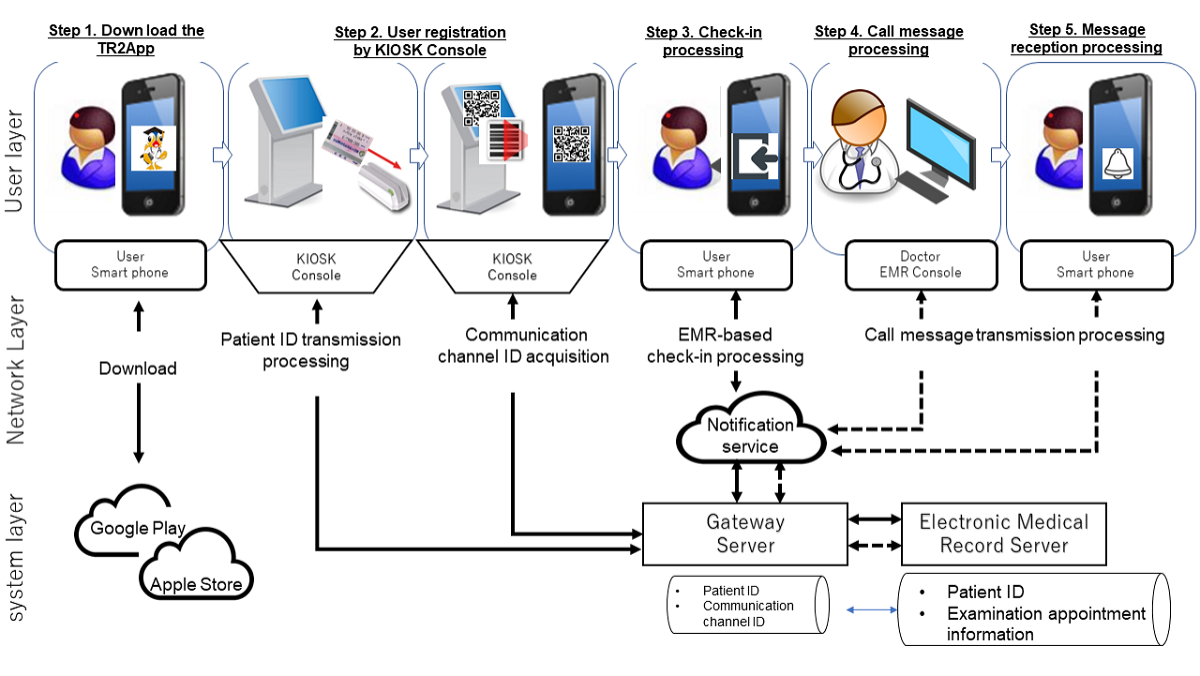
Schematic diagram of the Tori RinRin (TR2) system. EMR: electronic medical record.

The system processing that occurs from the time of app registration to the time of receiving examination call messages is shown in Figure 1.

#### Step 1: Download the TR2 App

First, outpatients downloaded and installed the examination guidance app (TR2 app) from the internet onto their mobile devices. The TR2 app can be downloaded for free from the Apple Store and Google Play.

#### Step 2: Communication Channel ID Acquisition

Outpatients used a barcode reader on a kiosk terminal in the hospital to read the patient ID number on their examination card. kiosk terminals are installed on all floors at which examination check-in is possible. The kiosk terminal sends the patient ID to a gateway server and acquires a communication channel ID. This communication channel ID, issued by Firebase Cloud Messaging (FCM) in the case of Android and by Apple Push Notification service (APNs) in the case of iOS, was used to send a text to the patient’s mobile device from their EMR via the internet [21,22]. The patient opened the examination guidance app and read the communication channel ID, which is displayed as a QR code on the kiosk terminal. This series of processes enables the linkage of data between the EMR console and the patient’s mobile device.

#### Step 3: EMR-Based Examination Check-in Using the Examination Guidance App

**Figure 2:**
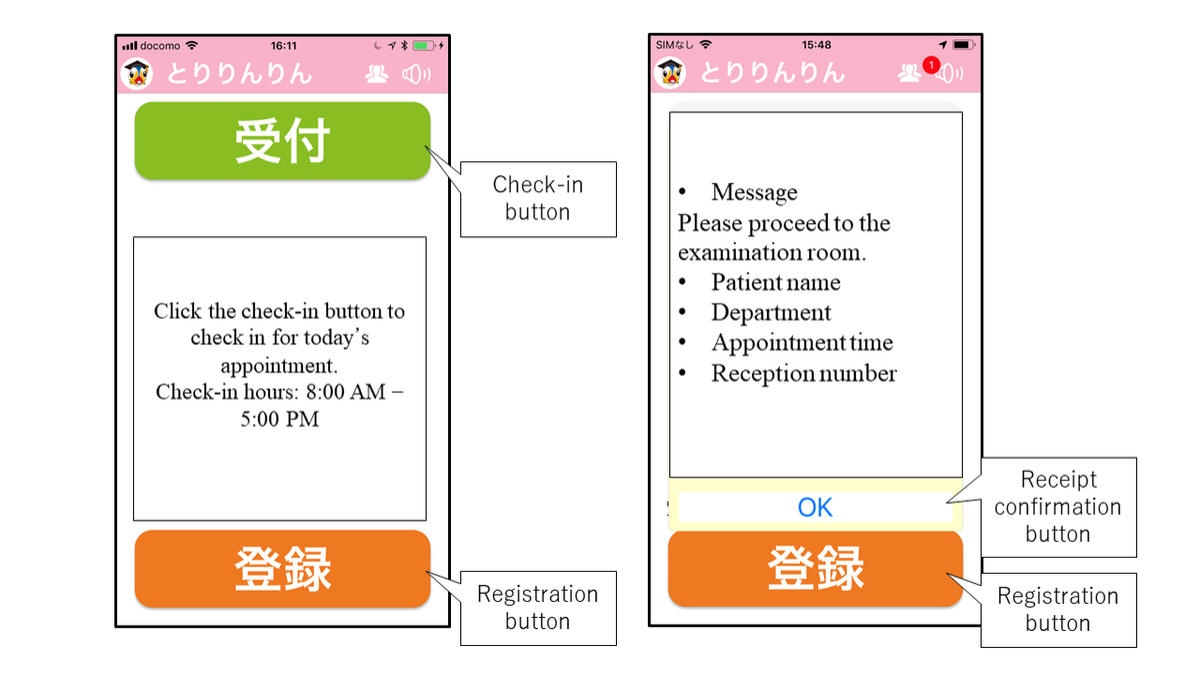
Example screens on the examination guidance app on the outpatient’s mobile phone.

When outpatients opened the examination guidance app, their location was identified by a GPS function, permitting them to check-in for their examination if they were within a 500-meter radius of TUH (Figure 2). When the outpatient tapped the check-in button on the examination guidance app, a message regarding their examination check-in was sent to their EMR via a gateway server, thereby concluding examination check-in. This operation allowed the physician to confirm that a patient scheduled for an examination that day had arrived at TUH.

#### Step 4: Transmission of the Examination Call Message From the EMR Console to the Examination Guidance App

**Figure 3:**
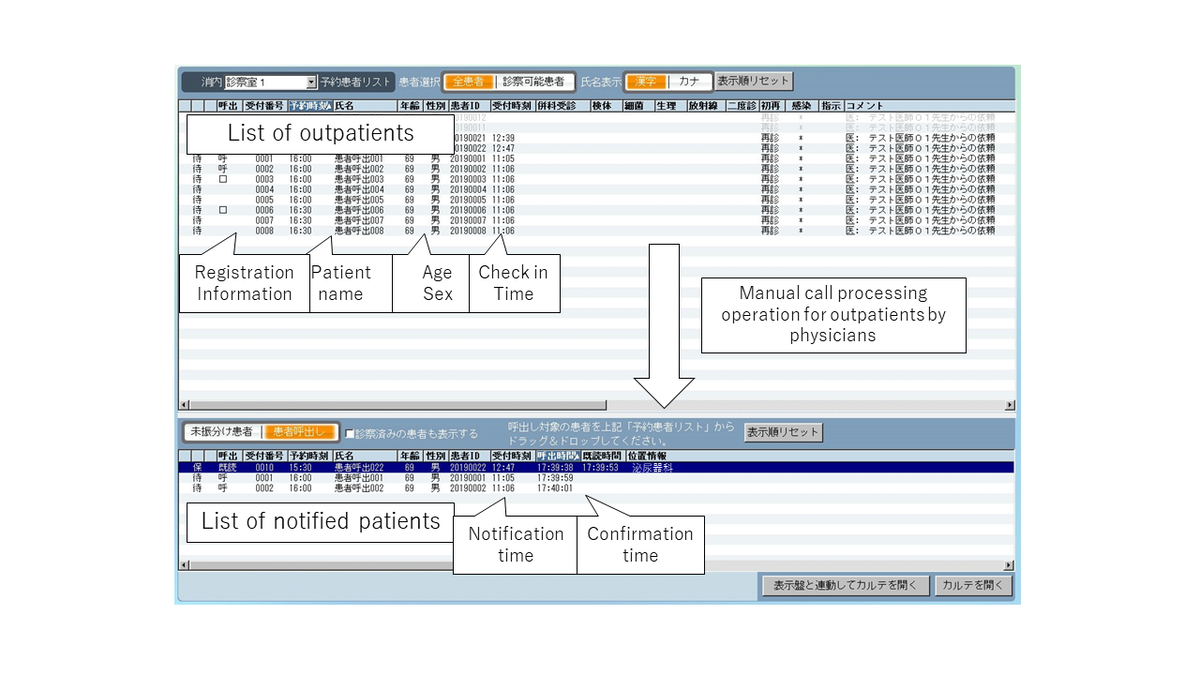
Electronic medical record (EMR)–based notification operation screen used by the physician.

The EMR console displays a list of patients scheduled for examination that day; when the physician selected a patient to call from that list, a notification was sent to the patient’s smartphone, calling the patient to the examination room (Figure 2). This notification operation is executed approximately 10 minutes before the examination begins. The message is sent via a gateway server as a push notification (Figure 3).

#### Step 5: Patient Response to the Call Message

Outpatients can receive guidance notifications on their smartphones from doctors before the start of medical examinations. When the patient presses the response button on the call message displayed on their smartphone, the response time is recorded in the “Confirmation time” column of the EMR (Figure 3).

### Components Comprising the TR2 System

Table 1 lists the components introduced to run the examination guidance app. The app can be used with both Android and iOS operating systems. To adapt the app to be used by a wide patient age range, the user interface was designed so that all operations can be completed simply by tapping buttons, without the need for flicking or typing. Confirmation operations from examination check-in (Step 3) to the examination call notification (Step 5) can be completed solely by tapping buttons. There were 4 kiosk terminals (1 on each floor) that were operated with a touch panel interface. As the kiosk terminal sent the patient’s ID (which is stored in the patient’s examination card) to the gateway server, it simultaneously acquired a communication channel ID from the gateway server. The gateway server has a mapping function to associate the patient ID used in the EMR with the communication channel ID and relays text messages to the patient’s smartphone via FCM/APNs.

**Table 1:** Components of the TR2 system.

Name	Use	Source	Hardware: operating system
Examination guidance app	Examination check-in, notification, and guidance	eBase Solutions	Android 8.0/iOS 11 or higher
Kiosk terminal	Data linkage	eBase Solutions	HP ProDesk 400 G6 SF: Windows 10pro
Gateway server	Message transmission, ID mapping	IBM Japan	Windows server 2016 Standard
EMR^a^ system	Examination records and patient notification operations	IBM Japan	IBM Power System E950, AIX

^a^EMR: electronic medical record.

### Postintroductory Evaluation of the TR2 System

To evaluate the usage of the TR2 system after its introduction, the following parameters were observed: (1) patient response time to the call notification function, (2) the number of registered medical guide apps, and (3) responses to an online questionnaire survey.

#### Patient Response Time

To evaluate the utility of the app-based messaging service, we investigated the time between notification transmission by the physician to confirmation of the message by the patient. Response time to calls was determined from data recorded in the EMR server database. The target data were collected from 363 people who used the TR2 system for 5 days from February 2, 2020 to February 7, 2020.

#### Number of Registered Medical Information Apps

From October 1, 2019 to September 31, 2020, we surveyed the number of newly registered patients in the TR2 system on a monthly basis to evaluate app usage. Additionally, from June 2020, as a countermeasure against COVID-19 infection, outpatients were encouraged to use the TR2 system to maintain social distancing. This COVID-19 countermeasure campaign was posted on a poster that recommended the registration and use of the TR2 system at outpatient reception areas of all medical facilities. We compared the utilization rate of the TR2 system in outpatients for the 3 months before and the 3 months after the COVID-19 countermeasure campaign was implemented. The utilization rate was calculated as the cumulative number of outpatients using the TR2 app during the 90-day period divided by the cumulative number of outpatients at TUH during the same period.

#### Evaluation of Convenience

To evaluate the convenience of the TR2 system, we conducted a questionnaire survey based on a 5-point scale. The question in this survey was “Did the TR2 system help reduce the burden of waiting time?” Respondents to the questionnaire were also able to voluntarily write comments. Respondents were selected by sending an electronic copy of the questionnaire form to patients who used the TR2 system for a period of 5 days from February 2, 2020 to February 7, 2020.

### Statistical Analysis

Statistical analyses were performed using R version 3.1.2. Differences in percentages between groups were compared using chi-square tests. *P* values <.05 were considered statistically significant.

## Results

### Time From Examination Call Message Transmission to Patient Response

We investigated the time from notification transmission by the physician to confirmation of the message by the patient. This investigation included 363 response times from 363 patients recorded from February 2, 2020 to February 7, 2020. The mean patient response time was 31 seconds, with a median of 14 seconds. Patients took ≥600 seconds to respond to notifications in 19 of 363 cases (5.2%). As a rule, physicians sent notifications 10 minutes before an examination was scheduled to start, and if patients failed to show up to the examination room by the start time, the physician could alter the examination queue.

### Changes in the Number of Registered Examination Guidance App Users

**Figure 4:**
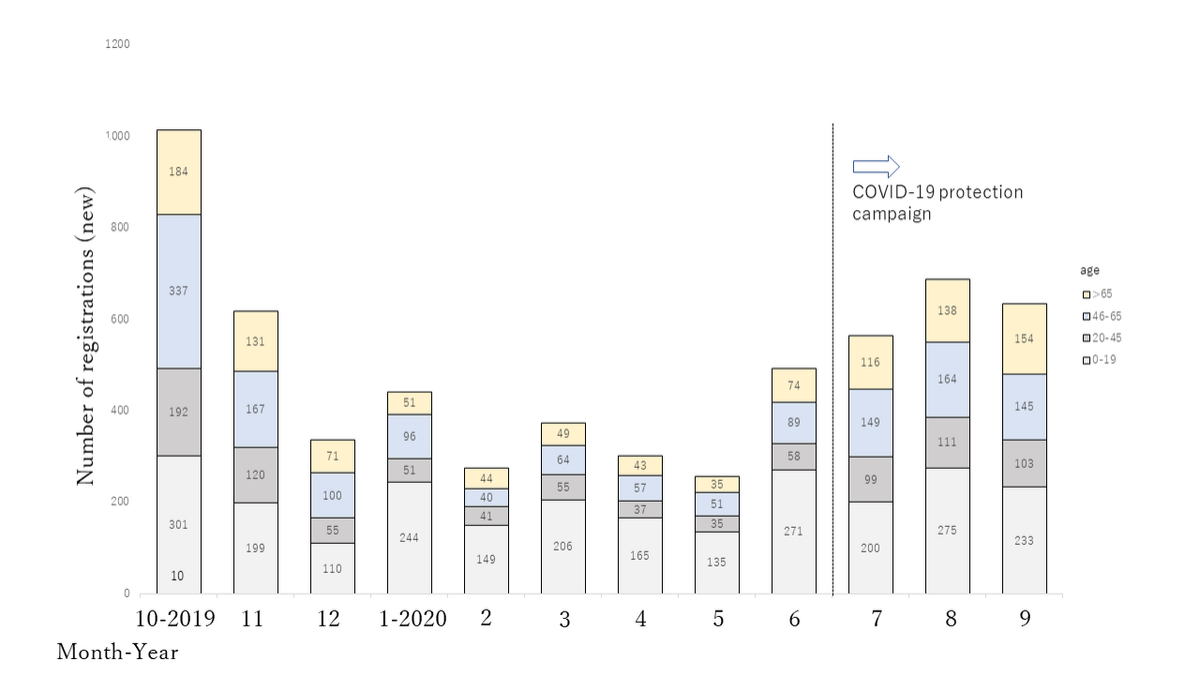
Changes in the number of registered examination guidance app users.

A total of 5994 patients registered for the examination guidance app from July 1, 2019 to September 30, 2020 (Figure 4).

From December 2019 to June 2020, the monthly mean number of new registered users was 353 (SD 81). In July 2020, TUH began to promote the use of the examination guidance app to prevent the spread of COVID-19. To assess the effect of promoting the examination guidance app usage as a measure to prevent COVID-19, we compared user registration rates before and after the introduction of the TUH COVID-19 prevention campaign. A comparison of the number of newly registered users for the 3 months before and after the start of the TUH COVID-19 prevention campaign revealed that the number of users increased 1.79-fold after the start of the campaign. Consequently, we found that app usage rate increased significantly from 16.4% (12,444 new registered users of 75,791 total outpatients) prior to campaign introduction to 17.7% (14,536 new registered users of 81,066 total outpatients) after campaign introduction.

### Results From the Anonymous Online Surveys of App Users

For the questionnaire survey for users, we decided to send a web-based, 5-point evaluation questionnaire to outpatients using the TR2 app. The number of respondents to the questionnaire was 166 of 363 (45.7%). The 5-point scale for assessing convenience consisted of the following responses: “Strongly agree,” “Agree,” “Neutral,” “Disagree,” and “Strongly disagree.” These responses were given by 82, 66, 15, 1, and 2 patients, respectively. From the results of the 5-point scale, we found that the TR2 system succeeded in its objective of reducing the burden imposed by wait times. Comments from the 41 patients who wrote in the free-response section of the survey were broadly divided into 3 categories (Table 2). From 41 respondents, 17 wrote favorably about the ability to wait for their examination at any location (such as in their cars or the cafeteria) and the ability to check-in for their examinations from outside the hospital. In contrast, the survey also revealed bugs such as notifications not being received by outpatients’ mobile devices.

**Table 2:** Classification of free-form questionnaire results about the Tori RinRin (TR2) system.

Category	Number of respondents	Responses
Reduction of stress imposed by wait times	17	Being able to wait where I wanted until my examination reduced my stress. Being able to check-in for my examination from outside the hospital was convenient. Being able to avoid crowded spaces while waiting for examinations can help to prevent COVID-19.
Opinions and requests regarding functions	16	I’d like to have an idea of how much longer I have to wait until my examination or how many people are waiting in front of me. They should let us check-in for examinations from more than 500 meters away. I want to know how many people are waiting in front of me so I can avoid COVID-19 exposure in crowded waiting rooms. They should add a page for blood tests and radiological tests. The text should be bigger, and the app should be easy to use.
Bugs in the app	8	I didn’t get an examination call message. The notification sound didn’t stop until I stopped it.

## Discussion

### Principal Findings

This study assessed the effectiveness of the TR2 system, which was developed to reduce the burden of outpatient waiting time, based on the system’s operation and user registration. The study determined patient response time to notifications regarding examination calls, changes in the number of registered users, and users’ assessments of the system, all of which revealed the advantages of introducing the system at a large medical facility as well as the associated problems in its implementation.

The examination guidance app’s call notification function employs push notifications using cloud messaging services (FCM and APMs). If the patient cannot receive examination call messages via push notifications normally, the outpatient examination queue will be disrupted, resulting in major inconvenience to the physician and patients. Our investigation of patient response times to messages revealed that approximately 5.1% of patients were unable to respond in fewer than 10 minutes; 10 patients reported that their examination notification messages were delayed. Push notifications can convey more information than other forms of notification such as phone calls and can reduce communications costs, which are advantages that have led many companies to adopt push notifications; however, they are highly dependent on individual smart devices. For example, in low power mode in iOS, when the device’s battery percentage falls below a certain level, push notifications are delayed [23]. We believe that the cause of 5.1% of delays in response to call messages were because of these smartphone-specific functionalities. One conceivable solution to such problems is to check for delays based on gateway server logs and switch from push notifications to phone calls or text messages when patients do not respond.

Regarding the number of registered examination guidance app users, approximately 12 months after the system was launched, about 6000 patients were registered for the TR2 system.

We learned that the change in the number of users was greatly affected by factors such as the app promotion campaign, COVID-19 pandemic, and outpatient age. Many apps developed to combat COVID-19 use iBeacon and GPS to enable determination of infected patients’ contact history and tracking of their actions [14,24-29]. In contrast, while our examination guidance app was not developed with the intention to combat COVID-19, it enables outpatients to wait in the parking lot or anywhere else until their examinations, thereby allowing them to avoid crowded waiting areas, reducing contact among patients and subsequently reducing COVID-19 exposure. As indicated by the results of this study, the number of examination guidance app users increased significantly following the promotion of the app as a COVID-19 countermeasure, suggesting that outpatients had a positive attitude toward using the examination guidance app to prevent the spread of COVID-19. Japan has not yet reported sufficient studies on cases of infection among patients in waiting rooms or on the causes of such infections. We also do not know how effective our examination guidance app is in preventing COVID-19. However, when medical facilities provide examination guidance apps free of charge, patients who are wary of COVID-19 can use their own judgment to avoid crowded spaces and wait elsewhere for their examinations. As revealed in our study, this allows patients to wait where they please and helps reduce their physical and mental stress.

### Comparison With Prior Work

Similar examination guidance apps, including the Outpatient Guidance System (OGS) developed by Baek et al [30] and the EasyHos System by Vorakulpipat et al [20], have been previously developed [31]. These systems also send a notification of the day’s outpatient schedule (stored in the hospital information system database) to the patient’s mobile device. Although the studies that introduced these systems introduced their functions and reported on user registration outcomes and improvements in hospital duties, they did not sufficiently assess how the systems notified patients. Although there are other methods of notifying patients, such as email and phone calls, the rates of patient response to these have not been examined. Therefore, push notifications, which were used in this study, are an effective form of communication for medical facilities, considering the system design and implementation of the TR2 system.

A guidance function that uses iBeacon based on an OGS can determine a patient’s location within a hospital. With these functions installed, an examination guidance app can determine the crowding of patients within a hospital (which was not performed in this study) and enable assessment of the app’s effects in preventing COVID-19 transmission.

Reported methods for examination guidance other than providing an app, include lending mobile devices to patients. With this method, the initial investment in hardware is extremely expensive. A single mobile device costs approximately US $400, while a management unit costs at least US $20,000. At TUH, where about 1500 outpatients are examined per day, lending out mobile devices would require approximately 1000 devices. Therefore, introducing this system at TUH was estimated to cost about US $420,000. If TUH had developed its own examination app, constructing the system (including the cost to revamp the EMR) would have cost US $120,000. As smartphones have recently exploded in popularity, many of our outpatients already own smart devices. When the initial investment and hardware management costs are also considered, providing an examination call function with an app is advantageous in terms of introduction costs. However, as stated earlier, the causes of defects in the system are difficult to investigate, and operation verification would be required whenever iOS or Android operating systems are updated. Introducing an examination guidance app requires consideration of these merits and demerits as well as consideration of the facility’s budget.

### Limitations

Clinical features such as sex and underlying disease of TR2 system users were not examined in this study. If the relationship between the clinical characteristics of users and the utilization rate is clarified, it will be useful information for medical institutions targeting specific diseases when considering introduction of this medical guidance app. By using the EMR of the Gateway server that comprises the TR2 system and the mapping information of the communication channel ID, it is possible to link to the medical care database and analyze the clinical characteristics of each user. In this study, the effects and problems of system introduction on the outpatient clinic have not been completely evaluated. These evaluations will be conducted by polling doctors and outpatient staff using questionnaires.

### Conclusion

In this study, we evaluated the introduction and usage record of the medical guidance application TR2 developed to reduce the burden of waiting time for outpatients in large-scale medical institutions. The utilization rate of the app by outpatients 1 year after introduction of the app was about 17.9%. According to the results of the anonymized questionnaire targeting app users, 88% of the users of this system reported a reduction in the burden of waiting time. In addition, by using the TR2 system, outpatients could wait away from crowded places until the start of their medical examinations, allowing the app to be used as an infection control measure in outpatient waiting areas. In the future, in order to prove its effectiveness as an infection control measure, it will be necessary to assess the location of the patient prior to the start of the patient's examination and the congestion of the waiting area outside of the outpatient examination rooms. The results of this study will be a useful index for medical institutions considering the introduction of medical guidance apps.

## Abbreviations

APNs: Apple Push Notification service
EMR: electronical medical record
FCM: Firebase Cloud Messaging
OGS: Outpatient Guidance System
TR2: Tori RinRin
TUH: Tottori University Hospital
